# Evaluation of Antioxidant Capacity, Protective Effect on Human Erythrocytes and Phenolic Compound Identification in Two Varieties of Plum Fruit (*Spondias* spp.) by UPLC-MS

**DOI:** 10.3390/molecules23123200

**Published:** 2018-12-04

**Authors:** Karen L. Hernández-Ruiz, Saul Ruiz-Cruz, Luis A. Cira-Chávez, Laura E. Gassos-Ortega, José de Jesús Ornelas-Paz, Carmen L. Del-Toro-Sánchez, Enrique Márquez-Ríos, Marco A. López-Mata, Francisco Rodríguez-Félix

**Affiliations:** 1 Departamento de Biotecnología y Ciencias Alimentarias, Instituto Tecnológico de Sonora, 85000 Ciudad Obregón, Sonora, Mexico; karen.heruiz@gmail.com (K.L.H.-R.); luis.cira@itson.edu.mx (L.A.C.-C.); lgassos@itson.edu.mx (L.E.G.-O.); 2 Centro de Investigación en Alimentación y Desarrollo. Av. Río Conchos S/N Parque Industrial, 31570 Cuauhtémoc, Chihuahua, Mexico; jornelas@ciad.mx; 3 Departamento de Investigación y Posgrado en Alimentos, Universidad de Sonora. Encinas y Rosales s/n, 83000 Hermosillo, Sonora, Mexico; carmen.deltoro@unison.mx (C.L.D.-T.-S.); enrique.marquez@unison.mx (E.M.-R.); francisco.rodriguezfelix@unison.mx (F.R.-F.); 4 Departamento de Ciencias de la Salud, Universidad de Sonora. Bordo Nuevo S/N, 85199 Ciudad Obregón, Sonora, Mexico; marco.lopezmata@unison.mx

**Keywords:** phenolic compounds, *Spondias* spp., UPLC-MS, antioxidant capacity

## Abstract

Plum edible part was used to obtained extracts by during a 4 h maceration process using three different solvents (ethanol, methanol and water) for the determination of total phenols and flavonoids, antioxidant capacity by (2,2′-azino-bis(3-ethylbenzothiazoline-6-sulfonic acid) diammonium salt (ABTS), 2,2-diphenyl-1-picrylhydrazyl (DPPH) and hemolysis inhibition in human blood assays. Subsequently, phenolic compounds were identified using ultra-performance liquid chromatography (UPLC-MS). The results indicated that the ethanolic extract of plum fruit being a good source of phenolic (12–18 mg GAE/g FW) and flavonoids (2.3–2.5 mg QE/g FW) content in both varieties of plum. Also, the fruits proved a good source of antioxidants as measured by DPPH and ABTS; likewise, plum aqueous extracts showed the highest protective effect on human erythrocytes with 74.34 and 64.62% for yellow and red plum, respectively. A total of 23 bioactive compounds were identified by UPLC-MS, including gallic acid, rutin, resorcinol, chlorogenic acid, catechin, and ellagic acid, and the antioxidant capacity can be attributed to these species. The edible part of plum contains compounds of biological interest, suggesting that this fruit has antioxidant potential that can be exploited for various technologies.

## 1. Introduction

Food production and consumption are among humankind’s greatest needs [1]. Interest in food production is increasing due to the growing production of free radicals and its relationship to the development of various diseases such as cancer, cardiovascular disease and chronic, degenerative diseases [2]. Thus, consumer interest in the consumption of natural foods and foods with bioactive compounds which can provide health benefits and act as preventive medicines has been increasing in recent years [3].

Recent studies have shown that a wide variety of vegetables are appreciated for their therapeutic potential and health benefits because of their high contents of bioactive compounds that can act as natural antioxidants [4]. Among these compounds are vitamin C, carotenoids, anthocyanins and phenols, and of these, phenols are of particular interest because of their important biological activities [5]. From a phytochemical point of view, members of the Anacardiaceae family are rich in secondary metabolites, especially phenolic compounds [6]. Plum (*Spondias* spp.) has been widely used for medicinal and therapeutic purposes; however, in Mexico, there are still wild populations of plum that have not been studied, and these populations represent a viable alternative for the development of new technologies [7].

Chromatographic techniques using different detectors such as UV light, diode array and fluorescence are commonly used for the identification and quantification of bioactive compounds in foods. In addition, mass spectrometry (MS) has proved to be a useful tool for research because it is applicable in the study of bioactive compounds [8]. This technique is mainly based on fragmenting molecules and evaluating their mass differences and is primarily used to identify, confirm and detect the presence and structure of bioactive compounds by quantifying the atoms and molecular fragments in the compounds [9]. Therefore, the combination of these two technologies has good sensitivity, high dynamic range and versatility. High- and ultrahigh-resolution analyzers are becoming increasingly popular for obtaining metabolomic profiles; ultra-performance liquid chromatography (UPLC) provides better separation for complex biological mixtures with shorter run times and using porous particles with diameters smaller than 2 mm results in greater peak resolution and sensitivity compared to standard chromatography (such as high-performance liquid chromatography, (HPLC) [10,11]. Hence, the aim of this study was to identify and evaluate the content of bioactive compounds (especially phenols and flavonoids) by UPLC-MS, evaluate the antioxidant activity and protective effect on human erythrocytes of plum extracts.

## 2. Results and Discussion

### 2.1. Total Phenolic and Flavonoid Contents

**Figure 1:**
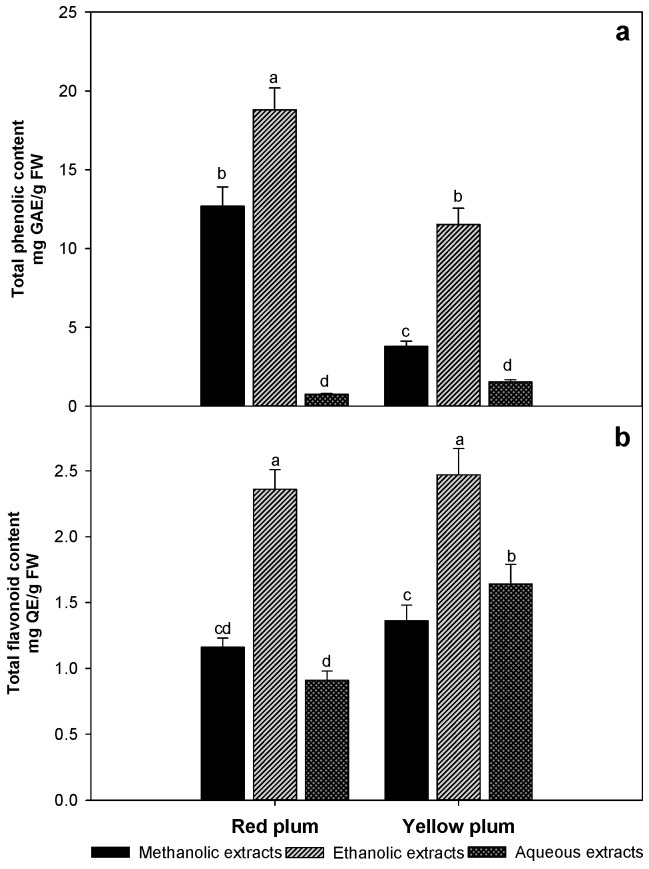
Total phenolic (**a**) and flavonoid (**b**) contents in methanolic, ethanolic and aqueous extracts of red and yellow plum variety. The data are the mean values of at least three determinations (n = 3) ± standard deviation (error bars). Bars with different letters are significantly different (*p* ≤ 0.05).

Phenolic compounds are the main secondary metabolites present in plants due to their protective effects in plants, and they could have similar effects in the human body; they can act as natural antioxidants, since they may reduce free radical formation caused by different types of stress [4,12]. The total phenolic contents obtained in the two plum varieties are show in Figure 1a, and the values ranged from 1 to 18 mg GAE/g FW. Red plum presented higher phenolic contents in its methanolic and ethanolic extracts, and significant differences were found for both the variety of plum and the organic solvent used. However, the results of the aqueous extracts were not significantly different, which can be attributed to the polarity of the solvent and compounds. The ethanolic extracts of both varieties showed the highest contents of phenolic compounds; therefore, ethanol was the optimal extraction solvent because it is an organic solvent and is not toxic to humans.

Other studies reported lower results than those obtained in this study. Murillo, Britton and Durant [4] obtained values of 1.88 and 1.08 mg GAE/g FW for red and yellow plum, respectively. Likewise, Zielinski, Ávila, Ito, Nogueira, Wosiacki and Haminiuk [13] reported values from 0.270 to 0.317 mg GAE/g using pulp from yellow and red plum. However, Bazílio-Omena, Barros-Valentim, Da Silva-Guedes, Rabelo, Mano, Henriques-Bechara, et al. [14] found higher values of 112 mg GAE/g DW for plum peel and similar values (13 mg GAE/g DW) for pulp; these results can be attributed to the phenolic compounds in fruits being concentrated in peels and seeds as a defense mechanism against stress. Several studies mention that the distribution of phenolic compounds is different between the seed, peel and edible parts of fruits [4,15].

Likewise, Figure 1b presents the results obtained for total flavonoid content, which ranged from 1 to 2.5 mg QE/g FW; when using ethanol as the extraction solvent yellow plum extracts presented greater values than those obtained with red plum (2.5 and 2.3 mg QE/g FW, respectively), but the difference was not significant (*p* < 0.05). Few studies have reported the identification and quantification of flavonoids in the different varieties of plum analyzed in this study. They have been reported values of flavonoids contents of 1.38 mg CE/g [13] and 0.38 mg of Q/g FW [16] for pulp of yellow plum. Other research also showed lower flavonoid values relative to the results obtained in this study, which may be due to the extraction procedure used or may be associated with the agronomic conditions and physiological factors of the fruit [17].

### 2.2. Antioxidant Capacity

**Figure 2:**
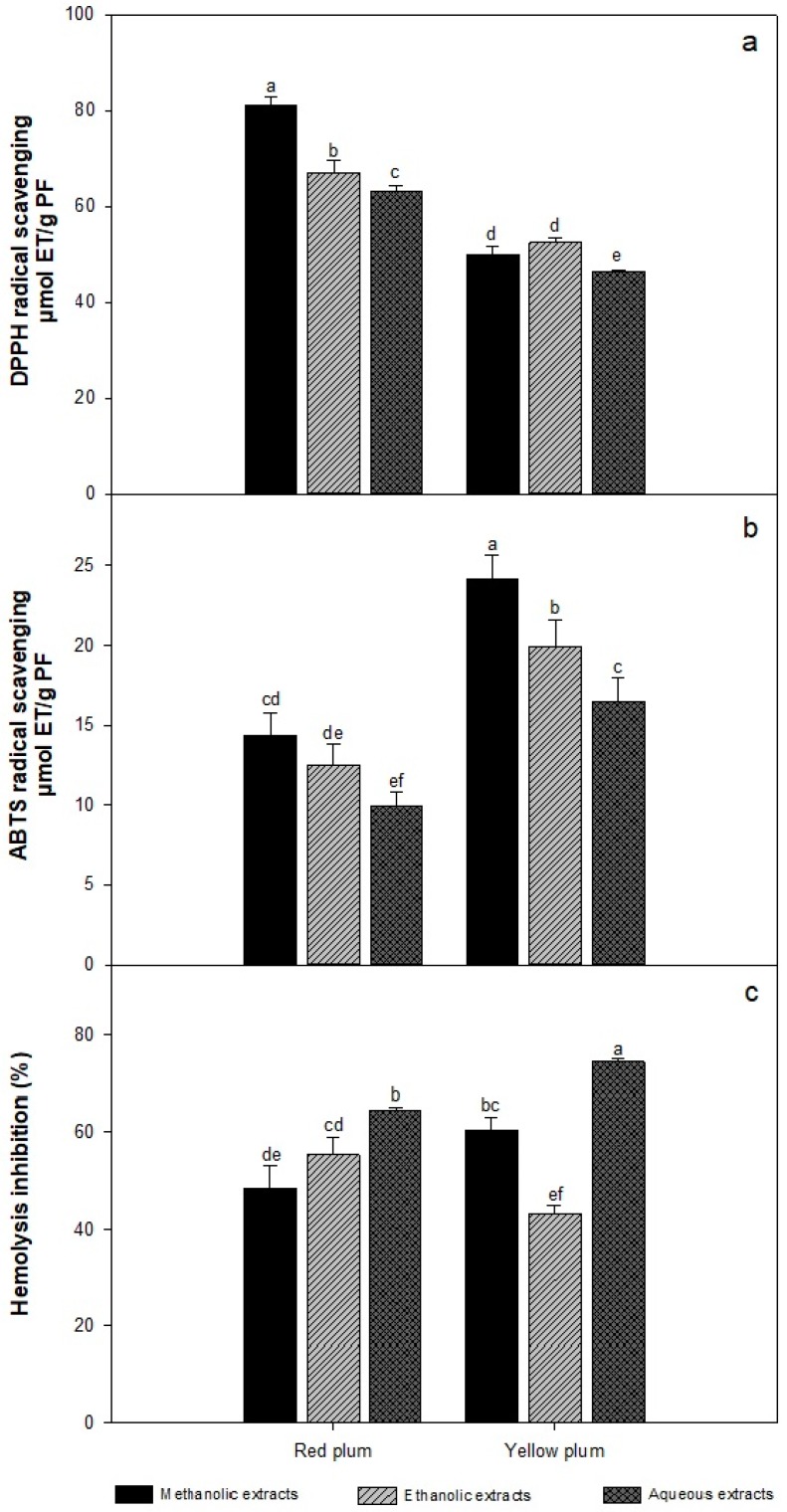
Antioxidant capacities of methanolic, ethanolic and aqueous extracts of red and yellow plum variety determined by DPPH (**a**), ABTS (**b**) and PHI (**c**) assays. The data are the mean values of at least three determinations n = 3) ± standard deviation (error bars). Bars with different letters are significantly different (*p* ≤ 0.05).

Figure 2a shows the antioxidant capacities of red and yellow plum extracts as determined by the DPPH method. Red plum presented higher capacities than yellow plum, with values from 60 to 80 μmol TE/g FW. The results show significant differences due to both the variety of plum and the choice of extraction solvent. In red plum, the methanolic extract gave the highest results, followed by the ethanolic extract and finally the aqueous extract. However, the same trend was not observed in yellow plum; the ethanolic extract had a higher antioxidant capacity than the methanolic extract, but the difference was not significant. The difference may be due to the polarity of the compounds present in the samples. Beserra-Almeida, Machado-de-Sousa, Campos-Arriaga, Matias-do Prado, de Carvalho-Magalhães, Arraes-Maia and Gomes-de Lemos [18] obtained lower values than those of the present study with approximately 1 μmol TE/g FW for yellow plum. Other authors reported obtained antioxidant capacities of 3.32 and 3.48 for red and yellow plum, respectively [16]. The variation between results can be attributed to the extraction method and solvents used in both studies. These authors used a sonication method that involved a shorter extraction time. Likewise, the differences can be attributed to the solvents used in those studies, which were mainly organic solvents such as acetone in an admixture with water and acetic acid; these solvents were not able to extract as much of the bioactive compounds.

Figure 2b shows the results obtained for the antioxidant capacities of the extracts based on ABTS radical inhibition. Yellow plum presented higher antioxidant capacities than red plum; the methanolic extract showed the highest values, followed by the ethanolic extract and finally the aqueous extract. 

The antioxidant capacities of the three extracts were significantly different, and the values from 15 to 25 μmol TE/g FW. The values of ABTS detected in the samples of our study, were higher than those of previous reports with values of 6 and 6.24 μmol TE/g FW for yellow plum [16,18]. The difference between these results can be attributed to the solvent used in the extraction, the affinity of the solvent for the phenolic compounds, and the extraction method. In general, the inhibition of free radicals can be attributed to the presence of phenolic compounds in the analyzed samples, and their activity depends mainly on the number and position of the hydroxyl groups on the aromatic ring of the phenol molecules; the inhibitory activity may also be affected by other factors such as the presence of glycosylated compounds, which may reduce the activity [19,20]. It is important to be considered that antioxidant capacity methods are used in order to give an idea about the quality and composition of the fruit [21]. Likewise, Prior, Wu and Schaich [22] recommended to use several methods to express the antioxidant capacity of a single food due to the diversity of antioxidant compounds they can contain. These authors also reported important differences between the mechanisms of the DPPH and ABTS assays. We choose to the determine the antioxidant capacity by the ABTS and DPPH assays, because the DPPH assay is very sensible for phenolic compounds, and phenols are the most abundant antioxidant compounds in mombins. Thus, the DPPH is ideal for mombins. However, other less polar antioxidants like carotenoids and tocopherols might be present, as occur in many other fruits rich in phenols, causing interference in the measurement by the DPPH assay. The interference of carotenoids in the DPPH assay has clearly been demonstrated [23]. On the other hand, ABTS assay works well for phenolic compounds and other less polar antioxidant compounds, like carotenoids [24]. This can be observed in our data of Figure 2a,b, where the bar for the metnanolic extract is consistently higher for both fruits with the ABTS assay, which does not occur with the DPPH assay.

Hemolysis causes erythrocyte rupture is a direct indicator of the damage caused by these radicals, and can be prevented by the consumption of antioxidants [25]. Therefore, the human erythrocyte protection assay has been used as a model to evaluate the oxidative damage to the biomembranes because they are highly susceptible to hemolysis. In Figure 2c, the results of the antioxidant capacity based on the human erythrocyte protection assay for each of the extracts analyzed are shown, and there is a statistically significant difference between different samples and different solvents. The extract with the highest PHI was the yellow plum aqueous extract followed by the aqueous extract of red plum with values of 74.34 and 64.62%, respectively. The results obtained in present study are similar to those obtained by [26] who reported values from 50 to 70% in their evaluation of quince leaves and green tea. However, Zhang, Hou, Ahmad, Zhang, Zhang and Wang [27] reported percentages lower than 50% in their evaluation of natural pigments. Likewise, this difference can be attributed to the fact that the matrices are different.

There are few studies related to the protective effects of erythrocytes, which may be due to the complications that can arise during the development of the technique. Other authors have reported that the protective effect toward erythrocytes may be related to the presence of bioactive compounds, such as phenols, flavonoids and carotenoids, in the samples, which can exert anti-inflammatory and antioxidant effects [25,28]. Nevertheless, in spite of the obtained results which showed high correlation between gallic acid and the protective effect on human erythrocytes in this study, it has been reported low correlation between phenolic compounds with in vivo methods [21]. Perhaps, this could be attributed to the fact that antioxidants have not decreased oxidative damage in vivo in some studies and the variation may be due to life habits, sex or genetics [29].

### 2.3. Identification of Bioactive Compounds by UPLC-MS

**Figure 3:**
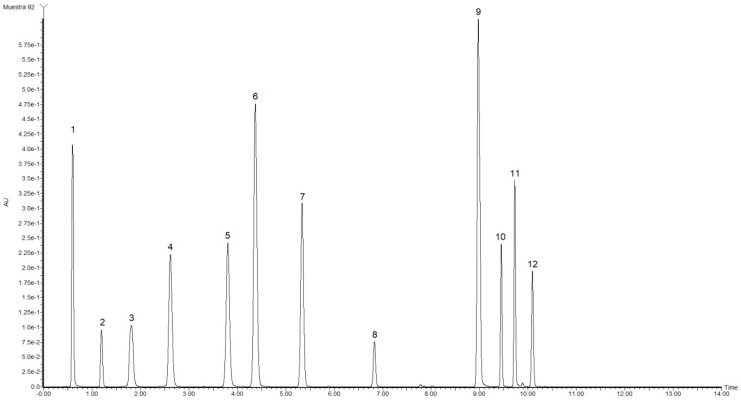
UPLC chromatogram of phenolic compound standards analyzed at 280 nm [y axis = intensity (absorbance unit, AU); x axis = retention time (min). Peaks: 1, gallic acid; 2, resorcinol; 3, chlorogenic acid; 4, caffeic acid; 5, vanillin; 6, coumaric acid; 7, ferulic acid; 8, rutin; 9, naringenin; 10, quercetin; 11, kaempferol and 12, eugenol.

Some phenolic compounds were identified and quantified by UPLC-MS in both plum varieties. Of these compounds, four were characterized based on a comparison of their retention times to those of standards (Figure 3), and their identities were confirmed by MS. However, 19 other bioactive compounds were identified by MS by comparison to other studies and based on the likelihood of occurring in the analyzed samples.

Table 1 shows the concentrations of phenolic compounds obtained in the red and yellow plums. The major compounds in the red plum methanolic extract were resorcinol, followed by chlorogenic acid, rutin and gallic acid, with values of 12.89, 11.15, 7.70 and 6.38 mg/100 g of extract, respectively. The difference between resorcinol and chlorogenic acid was statistically significant (*p* ≤ 0.05), but the difference between rutin and gallic acid was not significant.

The same trend was observed in the ethanolic extract, with values ranging from 3 to 5 mg/100 g of extract. The differences between resorcinol, rutin and gallic acid were significant, but the difference between chlorogenic acid and rutin was not. In yellow plum, the methanolic extract had the highest concentration of phenolic compounds, and resorcinol was the most abundant compound, followed by gallic acid, rutin and chlorogenic acid. The differences between all quantified compounds were all statistically significant. In the ethanolic extract, gallic acid was the major component, followed by rutin, resorcinol and chlorogenic acid, and the values ranged from 2.40 to 4.05 g/100 g of extracts. The difference between gallic and chlorogenic acid was significant, but the differences between other compounds were not.

**Figure 4:**
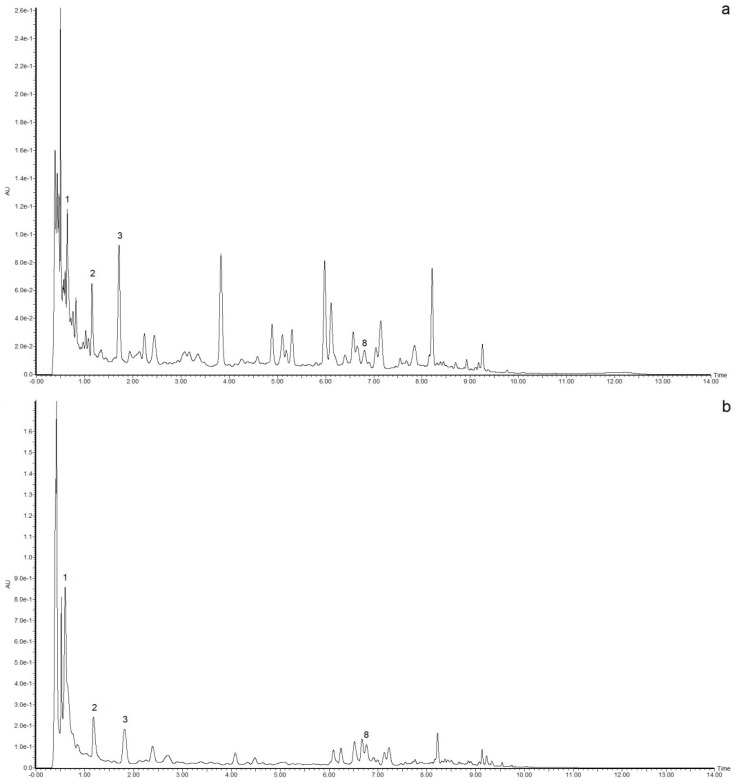
UPLC chromatograms of the methanolic extracts of the bioactive compounds of edible parts of red (**a**) and yellow (**b**) plums at 280 nm, [y axis = intensity (absorbance unit, AU); x axis = retention time (min)].

The results of the MS analyses are shown in Table 2, and the possible compounds that may be present in the samples are shown. Based on analogies to previous reports, vanillic acid, cyanidin, catechin and myricitrin were the components that presented the largest percent areas (from 3 to 7%) in the samples, and their retention times were 0.39, 0.51, 1.23 and 4.19 min (Figure 4), respectively. However, only catechin and myricitrin were present in both samples; thus, vanillic acid and cyanidin could be the main compounds responsible for the differences in the antioxidant capacities of the red and yellow plums. Other compounds that were presents in both samples are kaempferide, epigallocatechin, hesperetin and quercitrin. However, the compounds that were present only in red plum include luteolin, β-cryptoxanthin and ellagic acid [6,19,30]. Nevertheless, the area percentage is not indicative of if this compounds having an effect on the antioxidant capacity; it is only an indicator of their presence in the samples. To verify their presence in samples, standards of these compounds were also analyzed. Positive ionization mode is often used to ionize polar molecules; however, performing the sweep in both ionization modes is typically recommended to obtain unambiguous results of all the molecules present in the samples [10,31]. As shown in Table 2, all compounds were ionized by ESI (M + H)^+^, and only a few compounds appeared in ESI (M + H)^−^ hence, this ionization mode can be used for fragmentation of the molecular ion and the identification of compounds present in complex mixtures [8].

The compounds reported in other studies mainly include hydrolyzable tannins, phenolic acids, carotenoids and quercetin glycosylates, which elute first because they have large numbers of hydroxyl groups in their structures [6,32]. Most previous reports agree that acidification of the mobile phase can promote and improve the separation of phenolic compounds with weaker polarities; an aqueous mobile phase can be used to elute components slowly through the stationary phase, and organic solvents allow rapid elution of the components that are outside the stationary phase [33]. In addition, in UPLC analyses, the optimization of the chromatographic parameters, composition of the column and sensitivity of the equipment can be used to improves the resolution and sensitivity of the analytical method and reduce run times and solvent usage [34,35].

### 2.4. Correlation Between Bioactive Compounds and Antioxidant Capacity

Table 3 shows the correlation coefficients obtained between the antioxidant capacity values from the samples versus phenolic and flavonoid contents, as well as the content of phenolic compounds determined by UPLC-MS in the methanolic extracts. In red plum, the strongest correlation was for gallic acid, which showed values of 0.9952, 0.9608 and 0.9102 for ABTS, DPPH and the protective effect toward human erythrocytes, respectively. These values indicate that there is a strong relationship between the presence of gallic acid and the antioxidant activity of the methanolic extracts. For yellow plum, the total phenolic content was strongly correlated with antioxidant capacity determined by the ABTS method (0.9997); the correlation between the presence of resorcinol and the antioxidant capacity by the ABTS method was 0.9945; the correlation between the total flavonoid content and the ability to protect human erythrocytes was 0.9930. Thus, the strongest correlation was between the presence of phenolic compounds and the antioxidant capacity of the sample.

As shown in Table 4, in the ethanolic extracts, there is a strong relationship between the presence of rutin in the antioxidant activity by ABTS and hemolysis inhibition methods to red plum, with correlation coefficients of 1.0000 and 0.9854, respectively. This is unlike what was seen for the DPPH assay; in this case, the antioxidant capacity can be attributed to presence of chlorogenic acid. The trend in yellow plum was different due to the presence of chlorogenic acid and total flavonoids; gallic acid presented the highest correlations to ABTS, DPPH and hemolysis inhibition (0.9982, 0.9970 and 0.9999, respectively). Other studies found correlation coefficients of *r*^2^ > 0.9000, which indicated that the antioxidant capacity cannot be explained based by the contents of total phenolic compounds and flavonoids; therefore, the content of phytochemicals must be characterized to determine the active composition because it is necessary to know the structure of the molecules since the location and quantity of hydroxyl groups can influence the antioxidant capacity [19,36].

## 3. Materials and Methods

### 3.1. Reagents and Standards

All reagents and chemicals used were of analytical or HPLC grade. The solvents used for extraction and the sodium hydroxide were obtained from J. T. Baker (Ecatepec, Estado de Mexico, Mexico), as were the solvents used for chromatographic mobile phases, and Millipore water was obtained from Merck (Merck, Darmstadt, Germany). Sodium carbonate, Folin-Ciocalteu reagent, gallic acid, quercetin, aluminum chloride and sodium nitrate were from Sigma-Aldrich Chemical Co. (St. Louis, MO, USA). 2,2′-Azino-bis(3-ethylbenzothiazoline-6-sulfonic acid) diammonium salt (ABTS), 2,20-azobis(2-amidinopropane) dihydrochloride (AAPH) and 2,2-diphenyl-1-picrylhydrazyl (DPPH) radicals, and 6-hydroxy-2,5,7,8-tetramethylchroman-2-carboxylic acid (Trolox) were also from Sigma-Aldrich.

### 3.2. Sample Preparation and Extraction

In this study, the fruits were harvested from a family vegetable garden in Obregon, Mexico, transferred to the Emergent Technologies Laboratory at the Technological Institute of Sonora. The fruits were washed and the edible part were separated and stored in a freezer (−20 °C) until analysis.

Extraction of phenolic compounds was performed according to the method proposed by Suárez, et al. [37] with some modifications. A representative portion of fruits (8 g) was homogenized in 40 mL of water, water:ethanol (20:80, *v*/*v*) or water:methanol (20:80, *v*/*v*) at 250 rpm using ultra-turrax T18 basic homogenizer (Ika Works Inc., Wilmington, NC, USA). The water and the mixture water:ethanol were selected as solvents because these fruits are consumed as fruit water but their peel or the whole fruit might be used to obtain ethanol-based tinctures for therapeutic purposes, as reported for other small fruits [38]. The mixture water: methanol (20:80, *v*/*v*) is highly used for the efficient liberation of phenols from vegetable foods [39]. The extracts were vacuum filtered through Whatman #1 paper, the solvent was evaporated in a rotary evaporator (RE301, Yamato, Santa Clara, CA, USA) at 30 °C, and the residue was stored at 4 °C until further analysis. Six extracts were obtained for each fruit type (red and yellow mombin).

### 3.3. Content of Total Phenolic Compounds

The content of total phenolic compounds was determined by the method of Silva-Beltrán et al. [40] with slight modifications and adaptions to 96-well microplates. The reaction was performed by combining 150 μL of Folin-Ciocalteu reagent with 30 µL of extracts and 120 µL of sodium carbonate; the reaction was incubated at room temperature for 30 min, and the absorption was determined using a spectrophotometer microplate reader (Multiskan GO, Thermo Scientific, Waltham, MA, USA) at 750 nm. The content of total phenols was expressed as mg of gallic acid equivalents per g of fresh weight (mg GAE/g FW) using a calibration curve prepared with gallic acid as a standard.

### 3.4. Content of Total Flavonoids

The total flavonoid content was determined according to the procedure described by Zhang, et al. [41] with some modifications. First, 100 µL of extract was mixed with 430 µL of NaNO_3_ solution (95:5 *v*/*w*), and the solution was incubated for 5 min; 30 µL of AlCl_3_ (90:10 *v*/*w*) was then added, and the reaction was incubated for an additional 1 min and then mixed with 440 µL of NaOH (1 M). The absorbance was measured at 490 nm using a spectrophotometer microplate reader (Multiskan GO, Thermo Scientific, Waltham, MA, USA). A standard quercetin (Q) curve was prepared, and the total flavonoid contents are expressed as mg equivalents of quercetin per gram fresh weight (mg QE/g FW).

### 3.5. Trolox Equivalent Antioxidant Capacity (TEAC)

TEAC analysis was performed according to the procedure described by Re at al. [42] with modifications. The ABTS cation was generated using ABTS radical (0.019 g) dissolved in 5 mL of water with 88 µL of potassium persulfate solution (0.0378 g/mL); the mixture was incubated in the dark at room temperature for 16 h. Thereafter, 500 µL of ABTS radical was added to 30 mL of ethanol, and the absorbance was adjusted to 0.7 ± 0.02 nm using a microplate reader at 734 nm (Multiskan GO, Thermo Scientific, Waltham, MA, USA). The extracts (5 µL) were mixed with ABTS radical (295 µL) and incubated for 7 min at room temperature. Trolox was used as a standard, and the antioxidant capacities are expressed as micromoles of Trolox equivalents per gram fresh weight (µmol TE/g FW).

### 3.6. Radical Scavenging Capacity Using the DPPH Method

Free radical-scavenging capacity of the DPPH radical was measured according the procedure described by Moein and Moein [43] with some modifications. DPPH radical (0.0025 g) was prepared with 100 mL of methanol solution (80:20 *v*/*v*); the absorbance of the radical solution was adjusted to 0.7 ± 0.02 nm using a microplate reader at 490 nm (Multiskan GO, Thermo Scientific, Waltham, MA, USA). Thereafter, 20 µL of extracts were mixed with 280 µL of DPPH radical and incubated in the dark for 30 min at room temperature. The absorbance was read using a microplate reader at 490 nm, and the results are expressed as µmol TE/g FW.

### 3.7. Evaluation of the Protective Effect on Human Erythrocytes

To evaluate the protective effect on human erythrocytes, hemolysis was induced by AAPH radical according to the procedure described by López-Mata et al. [44] with some modifications. Erythrocytes were washed three times with phosphate-buffered saline solution (PBS) at pH 7.4, and then a suspension of erythrocytes was prepared with PBS (5:95 *v*/*v*). A mixture of erythrocytes (100 µL), extract (100 µL) and AAPH radical (100 µL) was prepared and incubated 3 h at 37 °C with continuous shaking (100 rpm). Then, 1 mL of PBS solution was added, and the mixture was centrifuged at 1500 rpm for 10 min. The absorbance of the supernatant was measured at 540 nm using a microplate reader (Multiskan GO, Thermo Scientific, Waltham, MA, USA). The results are expressed as a percentage of hemolysis inhibition (PHI) compared to a similar reaction without extract, and the value was calculated as follows:

(1) $$\mathrm{PHI}\;\left(\%\right)=\frac{\mathrm{AHI}-\mathrm{APE}}{\mathrm{AHI}}\times 100$$

where AHI = absorbance of hemolysis induced by AAPH; APS = absorbance of the plum extracts.

### 3.8. UPLC-MS Analysis of Bioactive Compounds

The separation of the phenolic compounds from the extracts of the edible parts of plum was performed using a Waters UPLC analytical system (Waters Corp. Singapure) equipped with a diode array detector coupled to a mass spectrometer. Additionally, the chromatography system was equipped with a vacuum degasser, an autosampler and a C_18_ analytical column (2.1 × 50 mm, 1.7 µm particle size; Acquity UPLC BEH). The phenolic compounds were identified according to the method proposed by Çam, İçyer and Erdoğan [45] with slight modifications. Three mobile phases were used to achieve compound separation: (A) 0.1 mL of acetic acid in 100 mL of deionized water, (B) methanol and (C) HPLC-grade acetonitrile. The flow rate for analysis was 0.3 mL/min; the column and sample temperatures were maintained at 35 °C and 20 °C, respectively; the injection volume was 1 µL; and the absorbance was monitored at 280 nm. The following gradient was used during the 14-min run: 0 min, 90% A, 5% B and 5% C; 6 min, 76% A, 12% B and 12% C; 11 min, 36% A, 32% B and 32% C; and 12 min, 90% A, 5% B and 5% C. The initial conditions were held for 15 min before each analysis.

Electrospray ionization (ESI) was operated in positive and negative mode, and spectra were acquired over a mass range of 100–750 *m*/*z* using a capillary voltage of 3.16 kV and a cone voltage of 30 V. The other optimum values of ESI-MS parameters were a desolvation temperature of 400 °C and a desolvation gas flow of 650 L/h.

### 3.9. Statistical Analysis

The experimental data were subjected to analysis of variance (ANOVA) for each analysis and multiple regression analysis using the StatGraphics by windows ver. 5.1 (Statgraphics Technologies, Inc. Virginia, USA). The results are expressed as the mean value ± standard deviation (SD). The ANOVA and significant differences were analyzed by Tukey’s multiple range test at a 0.95 (*p* < 0.05) confidence level. 

## 4. Conclusions

Red plum extracts presented higher content of phenolic compounds and antioxidant capacities in comparison with yellow plum. Ethanol was the best extraction solvent and can be used to obtain bioactive compounds. Nevertheless, yellow plum aqueous extract showed higher protective effect on human erythrocytes, followed by red plum aqueous extract. Additionally, approximately 20 bioactive compounds were identified by liquid chromatography coupled with MS; however, conclusive identifications were not reached in all cases, so standards had to be used to confirm the presence of the identified compounds. According with the results obtained in this study, the antioxidant activity may be strongly correlated with the presence of rutin and phenolic acids (gallic and chlorogenic). Finally, it should be considered that in vitro assays to evaluate antioxidant capacity are used to give an idea about the presence of bioactive compounds in food and it is important to carry out in vivo methods to evaluate their possible effect on human health.

## Figures and Tables

**Table 1:** Quantification of phenolic compounds in the extracts of the edible parts of red and yellow plum using two different solvents by UPLC-MS.

Compound	Red Plum (mg/100 g of Extract)		Yellow Plum (mg/100 g of Extract)	
	Methanol	Ethanol	Methanol	Ethanol
Chlorogenic acid	11.15 ± 0.73 ^b^	4.44 ± 0.34 ^b^	3.78 ± 0.07 ^d^	2.40 ± 0.09 ^b^
Gallic acid	6.38 ± 0.09 ^c^	3.11 ± 0.06 ^c^	6.82 ± 0.05 ^b^	4.06 ± 0.77 ^a^
Resorcinol	12.89 ± 1.06 ^a^	5.02 ± 0.63 ^a^	8.54 ± 0.68 ^a^	2.63 ± 0.62 ^ab^
Rutin	7.70 ± 0.19 ^c^	3.90 ± 0.17 ^b^	5.37 ± 0.50 ^c^	3.03 ± 0.01^ab^

The data are the mean values of at least three determinations (n = 3) ± standard deviation. Values in the same column with different letters are significantly different (*p* ≤ 0.05).

**Table 2:** Identification of possible bioactive compounds presents in extracts of the edible parts of red and yellow plum by UPLC-MS.

Bioactive Compound	Red (Rt)	Yellow (Rt)	MM (g/mol)	*m*/*z* (M + H)^+^	*m*/*z* (M + H)^−^	% of Area
Vanillic acid	0.39	N.D.	168	N.D.	167	7.00
Cyanidin	0.51	N.D.	287	288	286	6.33
Glycitein	0.51	0.57	284	285	N.D.	3.06
Catechin	1.23	1.25	290	291	289	3.93
Physcion	2.54	2.56	284	285	N.D.	2.24
Kaempferide	2.54	2.56	300	301	N.D.	2.24
3-Cafeoylquinic acid	3.50	N.D.	354	355	353	1.55
Myricitrin	4.19	4.23	464	465	N.D.	3.28
β-Cryptoxanthin	5.13	N.D.	552	553	551	1.98
Ellagic acid	5.55	N.D.	301	303	N.D.	1.59
Luteolin	6.30	N.D.	286	287	N.D.	2.63
Epigallocatechin	6.61	6.66	458	459	N.D.	1.48
Epigallocatechin gallate	6.61	N.D.	458	459	N.D.	0.97
Hesperetin	6.61	6.66	302	303	301	1.48
Quercitrin	6.77	6.84	448	449	447	2.10
Isorhamnetin	7.31	N.D.	302	303	N.D.	0.96
Baicalein/Galangin	7.31	N.D.	270	271	N.D.	2.33
Chalcone	8.26	8.3	208	209	N.D.	2.48
Quercetin	9.26	9.29	302	303	301	0.78

N.D. = Not detected; Rt = Retention time; MM = molecular mass; *m*/*z* = mass/charge. Engels et al. [6]; Cai et al. [19]; Silva et al. [30].

**Table 3:** Correlation between antioxidant capacity by the ABTS, DPPH and hemolysis methods with phenols, flavonoids and compounds quantified by UPLC-MS in methanolic extracts.

Bioactive Compounds	Red Plum			Yellow Plum		
	ABTS	DPPH	Hemolysis	ABTS	DPPH	Hemolysis
Phenols	0.8810	0.7800	0.6907	0.9997	0.8465	0.9686
Flavonoids	0.8130	0.6934	0.5812	0.9879	0.9096	0.9930
Chlorogenic acid	0.8581	0.7503	0.6461	0.9754	0.6922	0.8794
Gallic acid	0.9952	0.9608	0.9102	0.9520	0.6256	0.8338
Resorcinol	0.9139	0.8247	0.7337	0.8877	0.9945	0.9791
Rutin	0.8868	0.7878	0.6899	0.9520	0.6256	0.8339

**Table 4:** Correlation between antioxidant capacity by the ABTS, DPPH and hemolysis methods with phenols, flavonoids and compounds quantified by UPLC-MS in ethanolic extracts.

Bioactive Compounds	Red Plum			Yellow Plum		
	ABTS	DPPH	Hemolysis	ABTS	DPPH	Hemolysis
Phenols	0.9378	0.9857	0.8651	0.7794	0.8102	0.7257
Flavonoids	0.9997	0.8773	0.9812	0.9918	0.9970	0.9781
Chlorogenic acid	0.9099	0.9954	0.8261	0.9983	0.9941	0.9997
Gallic acid	0.9336	0.6286	0.9808	0.9972	0.9921	0.9999
Resorcinol	0.9828	0.9435	0.9370	0.7692	0.8007	0.7146
Rutin	1.0000	0.8660	0.9854	0.9039	0.9245	0.8660
